# A Method for Detecting LDoS Attacks in SDWSN Based on Compressed Hilbert–Huang Transform and Convolutional Neural Networks

**DOI:** 10.3390/s23104745

**Published:** 2023-05-14

**Authors:** Yazhi Liu, Ding Sun, Rundong Zhang, Wei Li

**Affiliations:** 1 College of Artificial Intelligence, North China University of Science and Technology, Tangshan 063210, China; liuyazhi@ncst.edu.cn (Y.L.); lw@ncst.edu.cn (W.L.); 2 Hebei Key Laboratory of Industrial Intelligent Perception, Tangshan 063210, China; 3 College of Management, North China University of Science and Technology, Tangshan 063210, China

**Keywords:** Low-Rate Denial of Service, Software-Defined Wireless Sensor Networks, Hilbert–Huang Transform, Convolutional Neural Networks

## Abstract

Currently, Low-Rate Denial of Service (LDoS) attacks are one of the main threats faced by Software-Defined Wireless Sensor Networks (SDWSNs). This type of attack uses a lot of low-rate requests to occupy network resources and hard to detect. An efficient detection method has been proposed for LDoS attacks with the features of small signals. The non-smooth small signals generated by LDoS attacks are analyzed employing the time–frequency analysis method based on Hilbert–Huang Transform (HHT). In this paper, redundant and similar Intrinsic Mode Functions (IMFs) are removed from standard HHT to save computational resources and to eliminate modal mixing. The compressed HHT transformed one-dimensional dataflow features into two-dimensional temporal–spectral features, which are further input into a Convolutional Neural Network (CNN) to detect LDoS attacks. To evaluate the detection performance of the method, various LDoS attacks are simulated in the Network Simulator-3 (NS-3) experimental environment. The experimental results show that the method has 99.8% detection accuracy for complex and diverse LDoS attacks.

## 1. Introduction

Software-Defined Wireless Sensor Networks (SDWSNs) introduce software-defined network architecture into wireless sensor networks, which equips sensor nodes and sink nodes with programmable functions in the control plane, realizing flexible control in sensor networks [1]. However, with the rapid development of SDWSNs, its security issues have also attracted increasing attention [2]. Due to the fact that the sensor nodes are low power and the wireless connections between the nodes are intermittent, based on this property of SDWSN, the Low-Rate Denial of Service (LDoS) attacks are able to launch elaborate attacks to make nodes unavailable.

Compared to Denial of Service (DoS) attacks, LDoS attacks are more harmful and more difficult to detect. Traditional DoS attacks involve a large number of data packets, which may cause anomalies in the statistical characteristics of network traffic to detect DoS traffic [3,4,5]. In contrast, LDoS attacks reduce the average network traffic, and attackers do not need to maintain a high attack rate. Instead, they periodically send the victim short burst traffics [6,7]. Therefore, a large-scale and long-term network paralysis can be caused by only a few attack packets, greatly reducing the throughput of victims. Additionally, a single LDoS attack flow disguised as a legally-formed pulse flow exhibits the same basic characteristics as normal traffic. Its average packet rate is low, 10–20% of normal data traffic, and it often submerges in normal traffic, making it difficult to be detected [8,9].

Currently, research mainly applies machine learning (ML) to extract attack traffic features in the network to detect and defend against attacks. For example, Deep Neural Network (DNN) models are highly effective in detecting attacks [10]. DNN models are trained using labeled traffic data and further used to classify traffic samples in the network. Thus, the ML approach is an appropriate choice to identify the network intrusions by acquiring traffic characteristics [11]. Due to the characteristics of LDoS attacks, traditional DNN models find it difficult to detect them [12,13,14]. However, detection methods based on the time–frequency domain can effectively identify LDoS attack features. Due to the role of Empirical Mode Decomposition (EMD), the Hilbert–Huang Transform (HHT) method in time–frequency transformations can extract the features of small-scale signals. EMD can adaptively perform time–frequency localized analysis, decompose data signals into a set of Intrinsic Mode Function (IMF) components, and extract meaningful instantaneous amplitude and frequency information [15]. However, in the process of decomposing the signal, if the signal has similar local features, which will generate similar IMF components in different decompositions, it may result in mode mixing, i.e., there may exist an overlap and a similarity between a set of IMF components [16].

Cutting-edge feature-based detection methods require significant computational resources and time for feature selection and model training, while time–frequency domain detection methods suffer from the detection of features in a small scale [17,18,19]. To fill the gap in detecting LDoS attacks using HHT-based spectral features, we propose the HCN method, which combines the Hilbert–Huang Transform and Convolutional Neural Network (CNN) methods to detect LDoS traffic. HCN optimizes the HHT with modal mixing to generate two-dimensional temporal–spectral features as the input feature vector and design a CNN model to classify attack traffic and normal traffic. The advantage of combining CNN with HHT lies in the ability of HHT on effectively capturing the time–frequency domain characteristics of LDoS traffic in the spectrum. By utilizing the advantages of deep neural networks in extracting features from two-dimensional spectrograms, CNN can extract covariant features related to data traffic from these spectrograms and classify the traffic data.

To evaluate the performance of the proposed HCN method, we used Mininet to build a network topology environment, and then carried out simulation experiments of SDWSN in NS-3. The traffic data came from the public dataset MAWI [20].

Specifically, we have made the following contributions:

- Redundant and similar IMFs in HHT were compressed to reduce the computation complexity and solve the modal mixing problem.
- Designed and implemented HCN, in which the compressed HHT was combined with CNN. HCN converted one-dimensional dataflow feature sequences into two-dimensional spectrogram features and then classified dataflows with CNN to improve the detection performance.
- Our simulated network environment was driven by real data traffic. The experimental results showed that the HCN was able to achieve an accuracy of 99.8%.

The remainder of this paper is organized as follows. Section 2 presents a review of related works. Section 3 introduces the proposed HCN method and the network structure of CNN. Section 4 describes the experimental setup and shows the results. Finally, this paper is summarized in Section 5.

## 2. Related Work

Kuzmanovic and Knighty first found LDoS attacks, and they proposed a new type of Low-Rate TCP-directed DoS attack in 2003 [21]. Since then, many researchers have begun studying the detection of LDoS attacks.

Currently, the detection of LDoS attacks can be divided into feature-based detection and time–frequency domain detection. Yan et al. [22] extracted the mean, variance, and entropy features of TCP traffic and employed them as features to train an enhanced logistic regression model for the purpose of detecting LDoS attacks. However, the feature extraction method used in this approach was relatively weak. Liu et al. [23] proposed a method for LDoS detection that utilized multiple feature fusions. Specifically, this approach extracted features from network traffic and, subsequently, conducted further processing on these features to fit a KNN classifier. However, this approach primarily relied on the KNN classifier for the detection of attacks, which is sensitive to noise data and can be easily affected by outliers. Zhang et al. [24] employed a combination of Principal Component Analysis (PCA) and Support Vector Machine (SVM) models for attack detection. This method filtered out noise interference, extracted the principal components of TCP flow characteristics, and trained the SVM model using the extracted training set principal components. However, this approach demonstrated limited efficacy in detecting complex attack behavior, despite its simplicity and efficiency.

Dan Tang et al. [17] introduced a LDoS attack detection method that employed a multi-feature fusion approach in conjunction with CNN. This method combined 17 distinct traffic features to generate a feature map that represented the network state. This feature map was then utilized as input to train the CNN model for effective attack detection. Expanding on this approach, they also advanced a LDoS attack detection scheme utilizing a Mean Shift clustering algorithm with a weighted Euclidean distance (WEDMS). The weighting factor was determined by the significance of the features [18]. Nevertheless, it required more intricate computations and more extensive model training.

The time–frequency domain detection approach is an effective method for detecting LDoS attacks. This method involves performing time–frequency analysis on network traffic data to extract essential features such as frequency, phase, and amplitude. These features are then meticulously analyzed and processed to identify the presence of LDoS attacks [25]. Agrawal et al. [13] proposed a method that employed power spectral density analysis to identify LDoS attacks in cloud environments. This method utilized Fourier Transform (FT) to transform time-domain data to a frequency–domain spectrum and calculated the values of the power spectral density. If the power spectral density values were concentrated in the low-frequency band, the traffic will be identified as an attack. The method proposed by Yue et al. [19] was a novel approach that combined Wavelet Transform (WT) and neural networks to accurately distinguish between normal traffic and LDoS attack traffic. This method extracted wavelet energy spectral coefficients at different time scales to analyze the multiple features of traffic and used a neural network to identify LDoS attacks. Fouladi et al. [26] proposed a scheme that combined Continuous Wavelet Transform (CWT) and CNN to detect network intrusions and defenses. This approach utilized features obtained from CWT as inputs of the CNN classifier, which distinguished attack samples from normal samples. Experimental results demonstrated that this scheme had a high identification rate for DNS amplification, NTP, and TCP-SYN flood attacks.

To clearly express the characteristics of each method, we listed the above methods in a table. Table 1 is a comparative analysis of the detection methods. Feature-based methods for detecting LDoS can identify the differences between normal traffic and attack traffic through machine learning and data mining. However, the selection of features and the training of models necessitate considerable computational resources and time. Thus, it requires less complex methods or models to detect various types of LDoS attacks. The shortcomings of the detection method based on the time–frequency domain are associated with the time–frequency transform method. Fourier transforms can only be applied to periodic signals. Therefore, it cannot effectively process non-periodic signals or signals with time constraints. Moreover, the selection of an appropriate wavelet basis function is critical for improving the accuracy of detection, and this varies depending on the type of signal.

HHT is an adaptive analysis method [27] that takes the multi-resolution analysis advantages of wavelet transforms while overcoming the difficulty in selecting wavelet basis functions. It can highlight non-stationary small signal characteristics produced by LDoS attacks and differentiate them from normal traffic. Therefore, we used HHT for time–frequency domain feature extraction to identify LDoS attacks. In HHT, similar IMF components are removed to effectively solve the mode mixing problem.

## 3. HCN Detection Method

**Figure 1:**
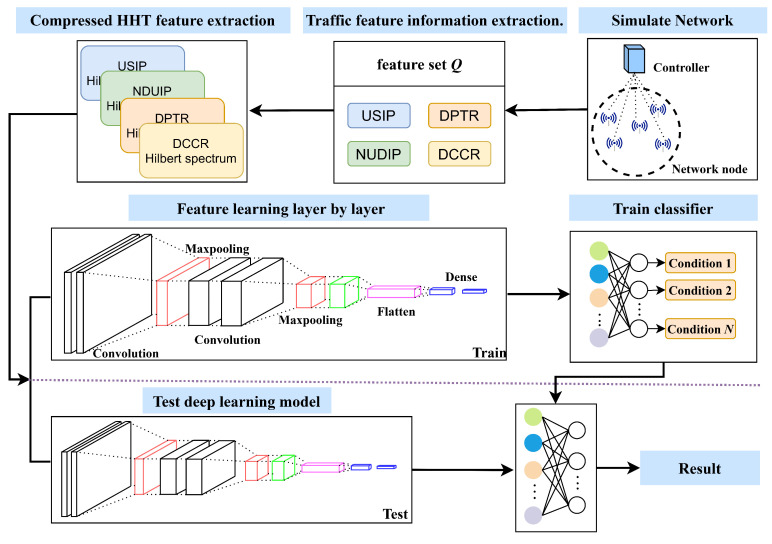
The flowchart of the HCN detection method shows that four features are extracted from the network nodes, and then HHT is performed on these features before inputting them to the CNN.

In this section, we introduce the HCN detection method. The flowchart of the proposed HCN method is shown in Figure 1, and the definitions of all the symbols are shown in Table 2.

### 3.1. Traffic Feature Information Extraction

For the purpose of detecting attack traffic, time series features were extracted from network nodes. We collected feature by extracting the total number of unique source IP addresses (USIP), the normalized number of total unique destination IP addresses (NUDIP), the differential packet transform rate (DPTR), and the differential network connection conversion rate (DCCR). The feature extraction followed the algorithm shown in Algorithm 1. The definitions of each feature are described in detail below:

**The total number of unique source IP addresses (USIP):** When the attacks are launched, the attacker sends a large number of packets with false IP addresses to attack other network nodes; thus, the value of USIP increases significantly. Therefore, USIP is adopted as the first feature to detect the attack.

**The normalized number of total unique destination IP addresses (NUDIP):** During the attack, the source IP addresses of the packets generated are random, but the destination IP addresses are set to the IP address of the victim node. Although the value of UDIP theoretically decreases, the change of UDIP is not obvious because other destination IP addresses also exist in the flow table. However, when normalized by the total number of packets, the value of UDIP changes significantly due to the dramatic increase in the total number of packets in the flow table. Therefore, normalized UDIP is adopted as the second feature to detect the attack.

**The differential packet transform rate (DPTR):** During the attack, the data packets within the network increase explosively. Thus, DPTR is applied as the third feature to detect the attack.

**The differential network connection conversion rate (DCCR):** Sometimes, there are elephant flows in normal traffic.The difference is that a normal elephant flow will not interrupt the connection request multiple times, whereas attack traffic will interrupt requests continuously. Therefore, DCCR is applied as the fourth feature.

The transformed two-dimensional spectrogram of these feature values can reflect the characteristic differences between normal traffic and attack traffic, which can be used to identify attack traffic.
**Algorithm 1** Traffic feature information extraction.**Require:** Network traffic data flow_t**Ensure:** Feature set *Q*
 1: **for** $\forall (Src_{\mathrm{ip}},Des_{\mathrm{ip}},P_{t},C_{t})\in flow\_t$ **do** 2:        **for** each time interval *t* **do** 3:              **if** $Src_{\mathrm{ip}}\to \exists$ **then** 4:                     $(Src_{\mathrm{ip}},count)\leftarrow count+1$ 5:              **else** 6:                     $(Src_{\mathrm{ip}},count)\leftarrow 1$ 7:              **end if** 8:              **if** $Des_{\mathrm{ip}}\to \exists$ **then** 9:                     $(Des_{\mathrm{ip}},count)\leftarrow count+1$ 10:              **else** 11:                     $(Des_{\mathrm{ip}},count)\leftarrow 1$ 12:              **end if** 13:              $\frac{P_{t+1}-P_{t}}{P_{t}}\leftarrow DPTR$ 14:              $\frac{C_{t+1}-C_{t}}{C_{t}}\leftarrow DCCR$ 15:              $(Src_{\mathrm{ip}},count)\leftarrow USIP$ 16:              $\frac{\left(Des_{\mathrm{ip}},count\right)}{P_{t}}\leftarrow NUDIP$ 17:        **end for** 18: **end for**

### 3.2. Compressed HHT

**Figure 2:**
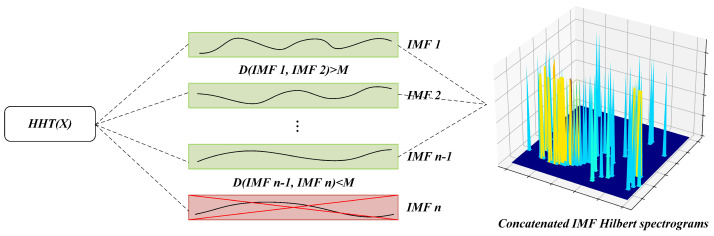
Compressed HHT. Green indicates retention, red indicates deletion, and the displayed three-dimensional plot shows the concatenated spectrogram.

During the process of decomposing the signal into IMF components using the HHT method, each IMF component was considered as a local feature of the signal. In applications, different signals may have similar local features, which could result in similar IMF components after the HHT decomposition. Although similar IMF components may not necessarily be caused by mode mixing; the local similarity of the data can also lead to this situation. However, it is undesirable regardless of the situation. Therefore, we calculate the Euclidean distance between adjacent IMF components to determine their similarity, as shown in Figure 2. To prevent feature duplication and save computational resources, IMF components with high similarity are directly removed, and the calculation of the next IMF component is stopped.

### 3.3. Frequency–Domain Feature Extraction

During a LDoS attack, the attacker injects a large amount of data traffic into the victim network in a short period of time until the network becomes congested. The attack traffic typically appears similar to normal traffic in the time domain, but exhibits low-frequency small signals in the frequency domain. HHT can extract the frequency–domain characteristics of such non-stationary small signals, enabling attack recognition.

To detect the small signal features of LDoS in the frequency domain, a feature sequence *X* is extracted from feature set *Q* in each time interval, as illustrated in Algorithm 2. A subsequence $X(t)=\{x_{t},\cdots ,x_{t+w}\}$ is obtained from sequence $X=\{x_{1},\cdots ,x_{w},\cdots ,x_{N}\}$ by a sliding window of length *w*. As attack traffic is bursty, each subsequence X(t) is non-stationary; as a result, it is difficult to find signal features in X(t). To resolve this issue, we use EMD to decompose the non-stationary time series into a set of linearly independent IMFs. Each IMF component represents the oscillations at different frequency bands of *X*. Then, we apply the Hilbert transform to each IMF component to obtain the instantaneous frequency and Hilbert spectrum, which include time, frequency, and amplitude component.
**Algorithm 2** Frequency–Domain Feature Extraction.**Require:** $[X_{1},X_{2},\cdots ,X_{n}]\in Q$**Ensure:** $S_{Q}$ 1: count ← 0 2: **for** $X_{i}$ **in** *Q* **do** 3:       **for** each time interval *t* **do** 4:             Statistics $\left(x_{t}\right)$ 5:             **if** count≤w **then** 6:                   $X\left(t\right)||x_{t}$; where || stands for concatenation. 7:                   count++ 8:             **else** 9:                   $X\left(t\right)\leftarrow x_{2}^{w}\left|\right|x_{t}$ 10:            **end if** 11:            $S_{X(t)}\leftarrow HHT\left(X\left(t\right)\right)$; where $S_{X(t)}\in R^{w\times w}$ 12:       **end for** 13:       $S_{{\left(X(t)\right)}_{t=1,\cdots ,n}}\in S_{Q}$; where $S_{Q}\in R^{w\times w\times n}$ 14: **end for**

A cubic spline function is used to fit the maximum envelope line for all local maximum points $e_{\max}\left(t\right)$ on the subsequence X(t). Similarly, the minimum points $e_{\min}\left(t\right)$ are identified, and their mean values, denoted as ml, are calculated as the average of the maximum and minimum envelope lines. Subtracting ml from the subsequence X(t) creates a new sequence C(t):

(1) C(t)=X(t)−ml

If C(t) satisfies the following conditions, it is the component of the first IMF [28].

1. In a local interval of the data, the number of extreme points of a function is equal to or differs from the number of zeros by, at most, one, and these extreme points and zeros appear alternately;
2. The average value of a function over the entire data range is zero;
3. The frequency of a function in a local interval varies monotonically with time.

(2) $$X\left(t\right)=\sum_{i=1}^{n}C_{i}\left(t\right)+r_{n}$$

$C_{i}\left(t\right)$ represents the *i*-th decomposed IMF component of X(t), while $r_{n}$ represents the *i*-th residual signal. To prevent mode mixing during the decomposition process, the Euclidean distance between adjacent IMF components is calculated to determine their similarity. The Euclidean distance *D* between the consecutive IMF components is calculated. If *D* is below a certain threshold, the newly decomposed IMF component is discarded and the calculation is stopped. If it is above the threshold, the process is repeated until all the IMF components are extracted. Figure 2 illustrates the process of decomposing X(t) into IMFs employing compressed HHT.

(3) $$D=\sqrt{{\left[C_{i}\left(t\right)-C_{i+1}\left(t\right)\right]}^{2}}$$

Subsequently, the retained IMF components are processed with Hilbert transform to generate the Hilbert spectrum of X(t). Then, the Hilbert spectra of IMF components are concatenated together to obtain H(ω,t).

(4) $$H\left(\omega ,t\right)=\sum_{i=1}^{n}a_{i}\left(t\right)e^{j\int \omega_{i}\left(t\right)dt}$$

where $a_{i}\left(t\right)$ and $\omega_{i}\left(t\right)$, respectively, denote the amplitude and instantaneous frequency.

**Figure 3:**
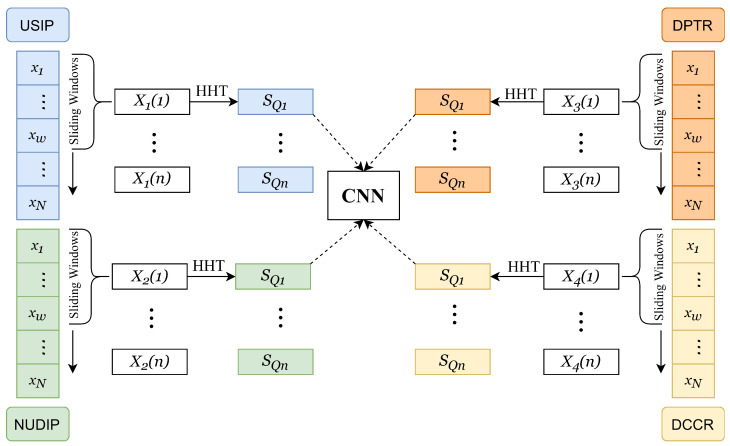
Compressed HHT-based feature transformation. The feature sequence *X* is passed through by a sliding window with a step size of 1. For each extracted subsequence, a Compressed HHT is applied, resulting in four sets of spectrograms. These spectrograms are then input to a CNN model.

Figure 3 illustrates the transformation of the feature set *Q* into four sets of two-dimensional spectra **$S_{Q}$** by compressed HHT. These four different combinations of spectral features provide a comprehensive presentation of the features and enhance the recognition ability of different features. Finally, the two-dimensional spectra are employed to train a CNN model and achieving the detection of attack traffic.

### 3.4. The HCN Model

**Figure 4:**
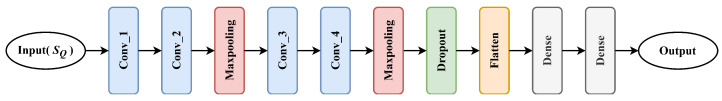
The HCN Model.

In this paper, a CNN is constructed by using a two-layer convolutional neural network and a single max-pooling layer, which is applied twice to the input of a two-dimensional spectrum, as depicted in Figure 4. The MaxPooling layer is utilized to further reduce the dimensionality of the information extracted by the convolutional layer, thereby improving the computational efficiency and enhancing the invariance of the image features. A Dropout layer is added to the Flatten layer to prevent overfitting during training of the model. The network uses 3 × 3 kernels in each layer, and the activation function used in all layers is ReLu. In the Dropout layer, each neuron has a 0.2 probability of being deactivated. Algorithm 3 describes the process of classification decision making by the HCN model.
**Algorithm 3** The HCN model for classification.**Require:** $S_{Q}$, two-dimensional spectrum.**Ensure:** ACC, accuracy of HCN classification. 1: **for** $i=1,2,\cdots ,N_{epochs}$ **do** 2:       **for** t=1,2,⋯,n **do** 3:             HCN ←${\left(S_{Q}\right)}_{t}^{N_{train}}$, training HCN models. 4:             Save training parameters. 5:       **end for** 6:       **for** t=1,2,⋯,n **do** 7:             HCN ←${\left(S_{Q}\right)}_{t}^{N_{test}}$, input testing dataset to the trained HCN model. 8:             ACC← HCN, calculating the accuracy of model judgments. 9:       **end for** 10:     Take the average of the accuracy rate of each epoch. 11: **end for**

## 4. Experiment

This section evaluates the performance of the HCN model in the SDWSN network. The experimental environment and the performance metrics are introduced, and the experimental results are analyzed.

### 4.1. The Network Topology

**Figure 5:**
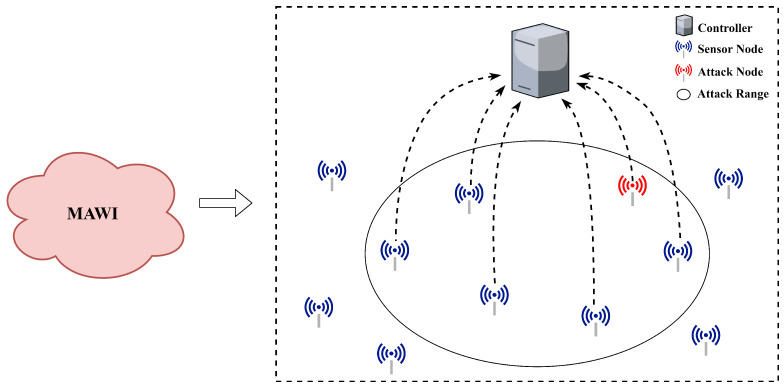
Network Topology.

To evaluate the performance of the HCN model, this paper employs the Mininet simulator to establish the network topology, which is then imported into NS-3 as the simulation environment for SDWSN. The experimental topology is shown in Figure 5.

### 4.2. Dataset

Since 2002, the MAWI laboratory has been committed to collecting and analyzing internet traffic data and has had a significant impact in this field [20]. We regenerated the network traffic from the MAWI dataset using the TcpReplay tool and rewrote the IP addresses based on the network node IPs in our experimental topology. The regenerated traffic was then injected into the network topology. Additionally, we generated attack traffic using the Hping3 and slowhttptest tools and sent it into the network from the attacker node in the topology.

The attack traffic in this paper can be classified into three types:

1. **HTTP slow DoS attack:** exhausts the resources of the target server by continuously sending incomplete or intentionally slow connection requests in order to achieve the attack purpose;
2. **ARP attack:** deceives other nodes in the network by changing the destination IP address of the traffic in the network to the victim’s IP address;
3. **Flood attack:** overloads the network and lowers its availability by sending a large amount of data traffic or control messages to the network.

The source IP addresses of all the attack data packets are fabricated or fake. From the victim’s perspective, the attack data packets appear to be coming from different sources. These attacks are able to push the victim into a congested state repeatedly.

**Figure 6:**
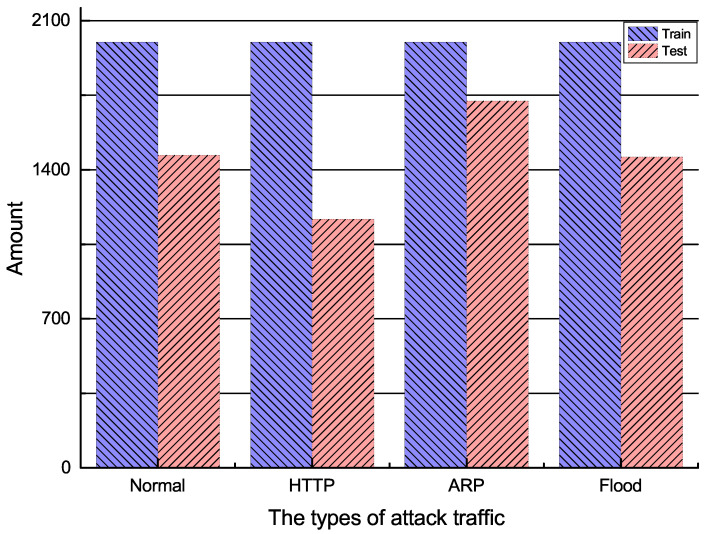
Number of training and testing sets for four types of traffic.

This experiment generated 4 h traffic, including 1 h normal traffic and 3 h attack traffic. We used the Wireshark software to capture the network traffic in the experimental topology. The feature set $Q=[X_{1},X_{2},X_{3},X_{4}]$ was collected and extracted for each time interval of t = 1 s in the network. A subsequence set $Q{\left(t\right)}_{t=1}^{13,800}=\left[{X_{1}\left(t\right)}_{t=1}^{13,800},{X_{2}\left(t\right)}_{t=1}^{13,800},{X_{3}\left(t\right)}_{t=1}^{13,800},{X_{4}\left(t\right)}_{t=1}^{13,800}\right]$ was obtained for each $X_{i}$ sequence in *Q* by a sliding window of length w=100. Subsequently, compressed HHT was performed on each subsequence $X_{i}\left(t\right)$ to obtain a three-dimensional feature spectrum. To facilitate deep learning in subsequent stages, the three-dimensional feature spectrum was projected and transformed into a two-dimensional frequency spectrum **$S_{Q}$**, resulting in 13,800 traffic samples, each containing four two-dimensional frequency spectrum. The training dataset consisted of 8000 labeled samples, including 2000 normal traffic samples, 2000 HTTP slow DoS attack traffic samples, 2000 ARP attack traffic samples, and 2000 Flood attack traffic samples. The testing dataset included a total of 5800 samples, which consisted of normal traffic and three types of attack traffic, as shown in Figure 6. The experimental parameters are shown in Table 3.

### 4.3. Experiment Results

**Figure 7:**
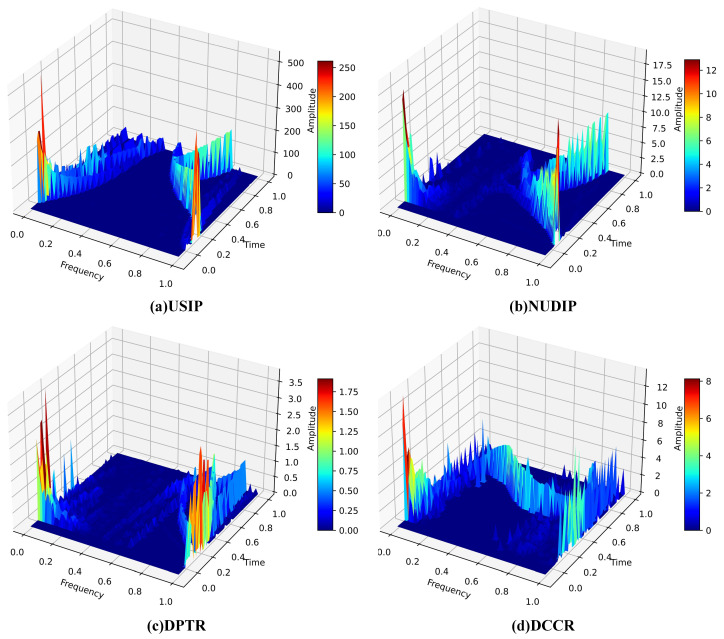
Three-dimensional spectrogram of selected traffic: (**a**) USIP. (**b**) NUDIP. (**c**) DPTR. (**d**) DCCR.

**Figure 8:**
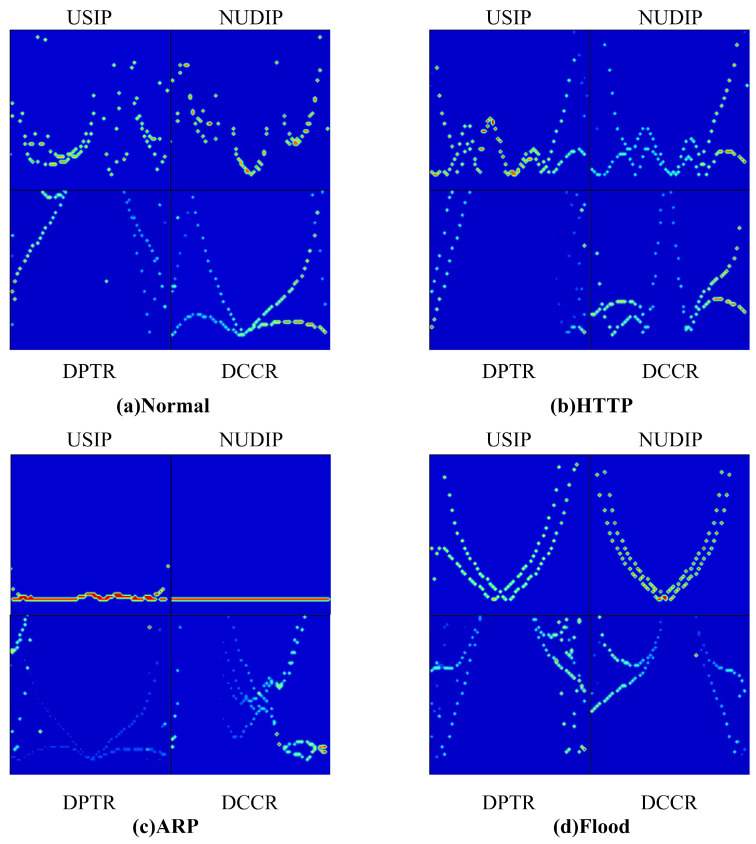
Two-dimensional spectrogram of three types of attack traffic and normal traffic: (**a**) Normal. (**b**) HTTP slow DoS attack. (**c**) ARP attack. (**d**) Flood attack.

Figure 7 shows the three-dimensional spectrogram of selected traffic, while Figure 8 shows two-dimensional spectrogram of three types of attack traffic and normal traffic. The time and frequency axes are normalized in the Figure. As shown in the Figures, the two-dimensional spectrum of each traffic type exhibits a distinct pattern. The spectrograms of different flows show different characteristics in frequency distribution and amplitude distribution. At low traffic rates, the frequency distribution of the spectrogram will be concentrated in the lower frequency range. However, for high traffic, the frequency distribution will expand to a higher frequency range, and the amplitude will also increase accordingly. Therefore, the HCN model employed four types of spectrograms as classification criteria to distinguish between normal traffic and attack traffic.

To evaluate the detection performance of HCN, experiments were conducted using FNr, FPr, and Accuracy as performance metrics, and compared with Multifractal [12], BP neural network [29], PSD [13], and MF-Adaboost [30]. PSD is a time–frequency domain detection method that provided 3.7% FPr and 4.9% FNr. Multifractal, BP neural network, and MF-Adaboost are ML-based detection methods. MF-Adaboost had the best detection performance, achieving 97.06% accuracy. HCN differed from these methods in that it combined time–frequency transforms with deep learning. HCN transformed the one-dimensional feature sequences into two-dimensional spectrogram features, thereby leveraging the advantages of deep neural networks in recognizing patterns in high-dimensional data spaces. According to the results presented in Table 4, the HCN method achieved low FNr and FPr rates while maintaining high accuracy. Principally, the FNr value of HCN was improved by an order of magnitude compared to the best algorithm mentioned above.

## 5. Conclusions

This paper proposed a LDoS attack detection method called HCN based on HHT and CNN. The proposed method involved the extraction of the features of multiple one-dimensional data sources from the network, and these were then transformed into two-dimensional frequency spectrum features by compressed HHT. This approach enabled a more comprehensive representation of network traffic characteristics. The resulting two-dimensional frequency spectrum features were input into a CNN for LDoS attack detection. Experiments were conducted in an SDWSN environment, and the results demonstrated that the HCN method achieved high accuracy while maintaining low false positive and false negative rates. Therefore, the method proposed in this paper was able to effectively detect LDoS attacks.

## Figures and Tables

**Table 1:** Summary of research status about detection methods.

Category	Proposal	Detection Method	Limitations
Detection methods based on features	Yan et al. (2019)	Enhanced logistic regression [22]	The feature extraction method was relatively weak.
	Liu et al. (2020)	KNN [23]	This method was sensitive to noise data and could be easily affected by outliers.
	Zhang et al. (2019)	PCA-SVM [24]	This approach demonstrated limited efficacy in detecting complex attack behaviors.
	Tang et al. (2020)	Multi-feature fusion [17]	They required intricate computations and extensive model training.
		WEDMS [18]	
Detection methods based on time–frequency domain	Agrawal et al. (2018)	FT [13]	FT could not effectively process non-periodic signals or signals with time constraints.
	Yue et al. (2018)	WT [19]	WT could not achieve high time and frequency precision simultaneously, and WT required the selection of an appropriate wavelet basis function.
	Fouladi et al. (2022)	CWT [26]	

**Table 2:** List of Notations.

Notation	Description
*Q*	Feature set
*t*	Sampling time interval
$x_{t}$	Feature Value
*D*	Euclidean distance
*M*	Euclidean distance threshold
*X*	Feature
*w*	Sliding window size
X(t)	The feature sequence after sliding window
*P*	The total number of packets
*C*	Total network connections
$e_{\max}\left(t\right)$	Local maximum points
$e_{\min}\left(t\right)$	Local minimum points
ml	The mean value between $e_{\max}\left(t\right)$ and $e_{\min}\left(t\right)$
C(t)	IMF component
$r_{n}$	Residual signal
H(ω,t)	The Hilbert spectra of IMF components
$a_{i}\left(t\right)$	Amplitude
$\omega_{i}\left(t\right)$	Instantaneous frequency
$S_{Q}$	Spectrogram after compressed HHT of *Q*
$N_{\mathrm{epochs}}$	Training rounds
$N_{\mathrm{train}}$	Training data set
$N_{\mathrm{test}}$	Testing data set

**Table 3:** Parameter list.

Parameter	Value
Frequency	100 HZ
Spectral resolution	64 × 64
Packets per second	200
Euclidean distance threshold *M*	1
Sliding window size *w*	100

**Table 4:** Comparative result.

Method	FNr	FPr	Accuracy
Multifractal	9%	10%	91%
BP neural network	3.32%	3.89%	96.68%
PSD	4.9%	3.7%	95.1%
MF-Adaboost	2.94%	0.33%	97.06%
**HCN**	**0.2%**	0.4%	**99.8%**

## Data Availability

No new data were created or analyzed in this study. Data sharing is not applicable to this article. As our data was generated by simulations, they lack the need to be shared.

## Abbreviations

LDoS	Low-Rate Denial of Service
SDWSN	Software-Defined Wireless Sensor Network
HHT	Hilbert–Huang Transform
IMF	Intrinsic Mode Function
EMD	Empirical Mode Decomposition
USIP	The total number of unique source IP addresses
NUDIP	The normalized number of total unique destination IP addresses
DPTR	The differential packet transform rate
DCCR	The differential network connection conversion rate
